# Pharmacological inhibition of DNA-PK stimulates Cas9-mediated genome editing

**DOI:** 10.1186/s13073-015-0215-6

**Published:** 2015-08-27

**Authors:** Francis Robert, Mathilde Barbeau, Sylvain Éthier, Josée Dostie, Jerry Pelletier

**Affiliations:** Department of Biochemistry, McGill University, Montréal, Québec, H3G 1Y6 Canada; Department of Oncology, McGill University, Montréal, Québec H3G 1Y6 Canada; The Rosalind and Morris Goodman Cancer Research Center, McGill University, Montréal, Québec H3G 1Y6 Canada

## Abstract

**Background:**

The ability to modify the genome of any cell at a precise location has drastically improved with the recent discovery and implementation of CRISPR/Cas9 editing technology. However, the capacity to introduce specific directed changes at given loci is hampered by the fact that the major cellular repair pathway that occurs following Cas9-mediated DNA cleavage is the erroneous non-homologous end joining (NHEJ) pathway. Homology-directed recombination (HDR) is far less efficient than NHEJ and makes screening of clones containing directed changes time-consuming and labor-intensive.

**Methods:**

We investigated the possibility of pharmacologically inhibiting DNA-PKcs, a key player in NHEJ, using small molecule inhibitors (NU7441 and KU-0060648), to ameliorate the rates of HDR repair events. These compounds were tested in a sensitive reporter assay capable of simultaneously informing on NHEJ and HDR, as well as on an endogenous gene targeted by Cas9.

**Results:**

We find that NU7441 and KU-0060648 reduce the frequency of NHEJ while increasing the rate of HDR following Cas9-mediated DNA cleavage.

**Conclusions:**

Our results identify two small molecules compatible for use with Cas9-editing technology to improve the frequency of HDR.

**Electronic supplementary material:**

The online version of this article (doi:10.1186/s13073-015-0215-6) contains supplementary material, which is available to authorized users.

## Background

The bacterial innate immune CRISPR (clustered regularly interspaced short palindromic repeat) system has emerged as a powerful molecular tool for genome engineering [1–4]. The key components of this system are a Cas9 endonuclease and a bifunctional single guide (sg) RNA. The sgRNA binds a DNA target site through sequence complementarity with the first approximately 20 5’ nucleotides whereas a 3’ aptameric domain is responsible for recruiting Cas9 to the genomic address [1]. The presence of an ^5’^NGG^3’^ protospacer adjacent motif (PAM) located immediately 3’ of the target sequence complement is the only essential feature of the target recognition site [5]. Cas9 will generate double-stranded breaks (DSB) at the target site which are repaired by the erroneous non-homologous end-joining (NHEJ) pathway to introduce indels (insertions/deletions) or if an appropriate target-homologous donor template is supplied *in trans*, by homology directed-repair (HDR) [6, 7].

NHEJ is initiated by a DSB leading to recruitment of a Ku70/80 heterodimer and the catalytic subunit of DNA-dependent protein kinase (DNA-PKcs). This tethers the two ends and a series of subsequent reactions result in resection, extension, and ligation [8]. In HDR, the DSB break is processed to a 3’ single-stranded overhang and a different set of repair proteins stimulate strand invasion by a donor homologous template, followed by repair [8]. In general, NHEJ is the more frequently observed repair pathway when using Cas9-mediated genome engineering, even in the presence of a donor HDR template [9–12]. Identifying cells of interest following HDR-directed editing is a labor-intensive and time-consuming process involving screening of individual clones to identify appropriate, correctly modified cells.

Improving HDR efficiency would significantly reduce the downstream workload involved in identifying appropriate clones of interest. Cell lines deficient for NHEJ components have significantly elevated levels of HDR suggesting competition between these two repair processes [13]. Hence suppression of the NHEJ key enzymes Ku70, Ku80, or DNA Ligase IV has been shown to stimulate Cas9-mediated HDR at the expense of NHEJ [14, 15]. A second strategy takes advantage of the fact that NHEJ occurs throughout the cell cycle whereas HDR-mediated repair is temporally restricted to late S and G2 phases [16]. Timed delivery of Cas9 protein and sgRNAs into nocodazole-mediated M phase synchronization of cells has been shown to increase HDR events [17]. Also, a recent unbiased screen of approximately 4,000 small molecules identified L755507 and Brefeldin A as capable of stimulating HDR, although the mechanism of action was not elucidated [18]. It has been recognized that DNA-PKcs can suppress HDR events following generation of a DSB [19]. The reliance of NHEJ on DNA-PKcs has prompted the assessment of DNA-PKcs mutants or small molecule inhibitors of DNA-PKcs on HDR following DSB induction – events that have been reported to increase HDR rates [20]. This led us to directly assess if pharmacological inhibitors of DNA-PKcs would be compatible with Cas9 editing tools and could be used to favor HDR repair events while restricting NHEJ-mediated mutagenesis events. We identified two small molecules compatible for use with Cas9-editing technology that improve the frequency of HDR while reducing NHEJ mutagenic events.

## Methods

### General methods

HEK293/17 (obtained from ATCC) and *Arf*^*−/−*^ MEFs (a kind gift of Dr. S. Lowe, Memorial Sloan Kettering Cancer Center) were maintained in DMEM supplemented with 10 % fetal bovine serum, 100 U/mL penicillin/streptomycin, and 2 mM glutamine. Plasmids were delivered to HEK293/17 cells by calcium phosphate transfection and to *Arf*^*−/−*^ MEFs by nucleofection using the Amaxa nucleofector I (Lonza, Walkersville, MD, USA). Plasmids pQCiG-Rosa, pQCiG-TLR, pQCiG-p53-1, pQCiG-p53-3, pLC-ROSA, or pLC-TLR have been described previously [12, 21]. The pCVL Traffic Light Reporter 2.1 and pRRL SFFV d20GFP.T2A.mTagBFP donor were purchased from Addgene. NU7441 and KU-0060648 were purchased from Selleckchem (Houston, TX, USA). Nutlin-3a was obtained from Sigma (St. Louis, MO, USA) and SCR7 was from Selleckchem (Burlington, ON, Canada). All compounds were resuspended in DMSO and stored at −80 °C. siRNAs targeting DNA-PKcs, PI3K-p110α, Ku70, Ku80, and the DNA Ligase IV mRNA were purchased from Dharmacon (Lafayette, CO, USA), resuspended in the company’s resuspension buffer to 10 mM and stored at −80 °C. For γ-irradiation, 293/TLR cells were plated at 25 % confluency and the next day were treated with DNA-PK inhibitors (2 μM NU7441 or 250 nM KU-0060648) for 1 h followed by 4 GY of γ-irradiation. After 30 min, the cells were harvested and extracts prepared and subjected to SDS-PAGE, followed by probing western blots using anti-eEF2 (Cell Signaling Technology; Beverly, MA, USA) and anti-p-H2AX (Upstate Biotechnology; Lake Placid, NY, USA). Compounds toxicity was determined using cell titer glow (Promega, Madison, WI, USA).

### TLR

The TLR assay was performed essentially as described by Certo *et al.* [22]. The presence of blue fluorescent protein (BFP) in the pRRL SFFV d20GFP.T2A.mTagBFP donor template plasmid allowed corrections for transfection efficiencies to be made. In all experiments, background fluorescence from non-transfected (<0.05 %) cells was subtracted from the values obtained from transfected cells. When reporting NHEJ efficiencies, we multiplied the value obtained by quantitating the mCherry^+^ cells by 3 since only one out of three repair events is expected to yield a ΔeGFP-T2A-mCherry fusion in the correct frame to generate mCherry^+^ cells. Transfections were performed in 6-well plates by the calcium-phosphate method using 2 μg of Cas9/sgRNA expression vector with 1 μg of donor plasmid or 0.1 μM donor oligonucleotide. Plasmids pcDNA-E1B55K and pcDNA-E4Orf6 were a kind gift from Dr. Phil Branton (Biochemistry Dept., McGill University, Montreal, QC, Canada). One microgram of pcDNA-E1B55K and pcDNA-E4Orf6, or of the pcDNA-3.1 control vector, were co-transfected with 2 μg of Cas9/sgRNA expression vector and 1 μg of donor plasmid. For siRNA experiments, 20 nM of each siRNA was transfected using lipofectamine following the manufacturer’s recommendations (Invitrogen, Carlsbad, CA, USA). Genome editing efficiency was determined by flow cytometry 5 days later. Knockdown efficiency was monitored by western blotting 48 h following transfections using antibodies directed to PI3K-p110α (Cell Signaling Technology; Beverly, MA, USA), DNA-PK (Cell Signaling Technology; Beverly, MA, USA), Ku70 (Santa Cruz Biotechnology, Santa Cruz, CA, USA), Ku80 (Santa Cruz Biotechnology, Santa Cruz, CA, USA), or DNA Ligase IV (Abcam Inc., Cambridge, MA, USA). Antibodies directed to adenovirus E1B55K and E4Orf6 were a kind gift from Dr. Phil Branton.

### Ion torrent sequencing

Following nucleofection of *Arf*^*−/−*^ MEFs with Cas9/sgp53 expression vectors, cells were allowed to recover for 16 h at which point 2 μM NU7441 or 250 nM KU-0060648 was added to the media followed by a 48 h incubation. Cells were then washed with PBS, media containing 5 μM Nutlin-3a added, and cells maintained for an additional 8 days. Genomic DNA was isolated by resuspending the cells in 500 μL DNA extraction buffer (0.2 % SDS, 5 mM EDTA, 200 mM NaCl, 100 mM Tris-HCl_8.5_, 50 μg Proteinase K) and incubating at 56 °C for 16 h. The DNA was recovered by precipitation with one volume of isopropanol, washed with 70 % EOH, air-dried, and resuspended in water. Selected genomic regions targeted by *sgp53-1* and *sgp53-3* were amplified using barcoded primers with engineered adaptor regions (Table 1) and Phusion HiFi polymerase (NEB; Beverly, MA, USA) using five cycles at an annealing temperature of 56 °C followed by 20 cycles at an annealing temperature of 68 °C. PCR products were purified using Ampure XP beads (Beckman; Fullerton, CA, USA) and the samples were quantified using the Quant-iT PicoGreen dsDNA Assay Kit (P11496, Life Technologies Inc.; Rockville, MD, USA), pooled in equimolar ratios and sequenced on an Ion Torrent Personal Genome Machine (PGM) as recommended by the manufacturer (Life Technologies; Rockville, MD, USA). Sequence reads were processed and analyzed as previously described [12].

**Table 1:** Oligonucleotide sequences used in this study

TLR donor sense	ACGGCAAGCTGACCCTGAAGTTCATCTGCACCACCGGCAAGCTGCCCGTGCCATGGCCCACCCTCGTGACCACCCTGACCTACGGCGTGCAGTGCTTCAGCCGCTACCCCG
TLR donor antisense	CGGGGTAGCGGCTGAAGCACTGCACGCCGTAGGTCAGGGTGGTCACGAGGGTGGGCCATGGCACGGGCAGCTTGCCGGTGGTGCAGATGAACTTCAGGGTCAGCTTGCCGT
p53-1 sense donor	CCCCAGGCCGGCTCTGAGTATACCACCATCCACTACAAGTACATGTGCAACAGTTCTTGTTGGGGGGCATGAACCGCCGACCTATCCTTACCATCATCACACTGGAAGAC
p53-1 antisense donor	GTCTTCCAGTGTGATGATGGTAAGGATAGGTCGGCGGTTCATGCCCCCCAACAAGAACTGTTGCACATGTACTTGTAGTGGATGGTGGTATACTCAGAGCCGGCCTGGGG
P53-1 BarcodeIonXpress_1	CCATCTCATCCCTGCGTGTCTCCGACTCAGCTAAGGTAACGATTTCACCTGGATCCTGTGTCT
P53-1 BarcodeIonXpress_3	CCATCTCATCCCTGCGTGTCTCCGACTCAGAAGAGGATTCGATTTCACCTGGATCCTGTGTCT
P53-1 BarcodeIonXpress_4	CCATCTCATCCCTGCGTGTCTCCGACTCAGTACCAAGATCGATTTCACCTGGATCCTGTGTCT
P53-1 BarcodeIonXpress_6	CCATCTCATCCCTGCGTGTCTCCGACTCAGCTGCAAGTTCGATTTCACCTGGATCCTGTGTCT
P53-1 BarcodeIonXpress_8	CCATCTCATCCCTGCGTGTCTCCGACTCAGTTCCGATAACGATTTCACCTGGATCCTGTGTCT
P53-1 BarcodeIonXpress_10	CCATCTCATCCCTGCGTGTCTCCGACTCAGCTGACCGAACGATTTCACCTGGATCCTGTGTCT
P53-1 barcoding Reverse oligo:	CCTCTCTATGGGCAGTCGGTGATGTCCCTCCAATTTTACACCT
p53-3 sense donor	CCACACCTCCAGCTGGGAGCCGTGTCCGCGCCATGGCCATCTACAAAAAGTCCCAACAATGACGGAGGTCGTGAGACGCTGCCCCCACCATGAGCGCTGCTCCGATGGT
p53-3 antisense donor	ACCATCGGAGCAGCGCTCATGGTGGGGGCAGCGTCTCACGACCTCCGTCATTGTTGGGACTTTTTGTAGATGGCCATGGCGCGGACACGGCTCCCAGCTGGAGGTGTGG
P53-3 BarcodeIonXpress_38	CCATCTCATCCCTGCGTGTCTCCGACTCAGTGGAGGACGGACGATACGTGCCCTGTGCAGTTGTG
P53-3 BarcodeIonXpress_39	CCATCTCATCCCTGCGTGTCTCCGACTCAGTAACAATCGGCGATACGTGCCCTGTGCAGTTGTG
P53-3 BarcodeIonXpress_40	CCATCTCATCCCTGCGTGTCTCCGACTCAGCTGACATAATCGATACGTGCCCTGTGCAGTTGTG
P53-3 BarcodeIonXpress_41	CCATCTCATCCCTGCGTGTCTCCGACTCAGTTCCACTTCGCGATACGTGCCCTGTGCAGTTGTG
P53-3 BarcodeIonXpress_42	CCATCTCATCCCTGCGTGTCTCCGACTCAGAGCACGAATCGATACGTGCCCTGTGCAGTTGTG
P53-3 BarcodeIonXpress_43	CCATCTCATCCCTGCGTGTCTCCGACTCAGCTTGACACCGCGATACGTGCCCTGTGCAGTTGTG
P53-3 barcoding Reverse oligo:	CCTCTCTATGGGCAGTCGGTGATAGGGCTTACCATCACCATCG

## Results and discussion

**Fig. 1:**
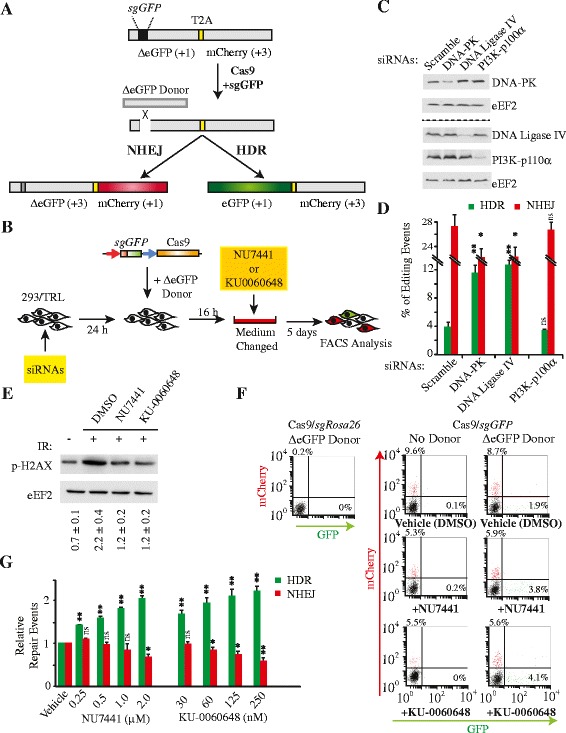
DNA-PKcs inhibitors stimulate HDR following Cas9 induction of a DSB. **a** Schematic outline of the Traffic Light Reporter assay. The eGFP and mCherry open reading frames (ORF) are fused by a ribosome skipping sequence (T2A) where translating ribosomes skip from a Gly to a Pro codon without forming a peptide bond while maintaining the reading frame to produce two distinct polypeptides [39, 40]. In the TLR, the eGFP and mCherry ORFs are positioned in different frames and NHEJ repair of a DSB directed to the GFP ORF will, in one out of three events, place ΔeGFP in frame with mCherry - leading to mCherry production. Since the eGFP ORF contains an insertion harboring a premature termination codon (and lacks a start codon), its expression can only be recovered upon HDR-mediated repair following delivery of an appropriate homologous template. The reading frame for each fluorescent protein is indicated in parentheses. **b** Outline of siRNA and drug treatment protocol. In one set of experiments, the 293/TLR cell line was transfected with the indicated siRNAs (*yellow box*) and 24 h later expression constructs driving synthesis of Cas9 and sgRNAs and an eGFP donor template were introduced into the cells. In a second set of experiments, the 293/TLR cell line was first transfected with expression constructs driving synthesis of Cas9 and sgRNAs and an eGFP donor template. Compounds were added 16 h later (*orange box*). In both cases, cells were allowed to propagate for 5 days before FACS analysis. **c** Knockdown of DNA-PK, DNA Ligase IV, and PI3K-p110α in 293/TLR cells transfected with the indicated siRNAs. Western blots were probed with antibodies to the indicated proteins. The dashed line separates two different sets of membranes. **d** Quantitation of genome editing events in 293/TLR cells transfected with the indicated siRNAs. *N* = 4; error bars represent S.D. Results are from biological replicates performed in technical duplicates. Significance (relative to scrambled siRNAs) was calculated using the Student’s *t*-test: *P ≤0.05; **P ≤0.01; ns, not significant. **e** DNA-PK inhibitors prevent H2AX phosphorylation upon γ-irradiation. Cells were pre-incubated with the indicated small molecules for 1 h followed by 4 GY of γ-irradiation (IR). Extracts for western blotting were prepared from non-irradiated (IR) cells (lane 1) or IR-exposed cells incubated in the presence of vehicle (lane 2), 2 μM NU77441 (lane 3), and 250 nM KU-0060648 (lane 4). Western blots were probed with antibodies directed to the proteins indicated to the left of the blot. The ratio of p-H2AX and eEF2 band intensities is indicated below the blots. **f** Flow cytometry analysis of 293/TLR cells transfected with pQCX vectors driving synthesis of Cas9 and sgRNAs targeting *Rosa26* or *eGFP*. When indicated, the GFP repair template, pRRL SFFV d20GFP.T2A.mTagBFP (ΔeGFP Donor) was also included. NU7441 and KU-0060648 were used at a final concentration of 2 μM and 250 nM, respectively. Cells were gated for the live population as determined by PI staining. Transfection efficiencies were greater than 70 %. **g** Quantitation of genome editing events from cells propagated in the presence of the indicated concentrations of DNA-PKcs inhibitors. *N* = 4; error bars represent S.D. Results are from biological replicates performed in technical duplicates. Significance (relative to vehicle) was calculated using the Student’s *t*-test: *p ≤0.05; **p ≤0.01; ns, not significant

To simultaneously monitor NHEJ and HDR repair processes following induction of a DSB, we utilized the convenient and robust ‘Traffic Light Reporter’ (TLR) assay developed by Scharenberg and colleagues [22]. This system relies on HDR-mediated repair to generate functional enhanced green fluorescent protein (eGFP) and NHEJ to enable mCherry production (Fig. 1a). We sought to validate our assay conditions for reporting on HDR and NHEJ events following suppression of DNA Ligase IV or DNA-PKcs using siRNAs, since suppression of these have been previously shown to increase HDR levels [22–24]. HEK 293 T/17 cells transduced with the TLR reporter (hereafter referred to as 293/TLR) were first transfected with either a scrambled siRNA or siRNAs targeting DNA-PKcs or DNA Ligase IV (Fig. 1b). Twenty-four hours later, Cas9/sgRNA expression vectors targeting the TLR eGFP cistron (*sgGFP*) in the presence of an eGFP homologous repair template were introduced (Fig. 1a, b). Flow cytometry analysis revealed that HDR occurred in about 4 % of the cells while NHEJ occurred in about 25–30 % of the cell population (Fig 1c, d). The analysis also revealed that knockdown of DNA Ligase IV or DNA-PKcs stimulated HDR approximately three-fold while reducing NHEJ (Fig 1c, d). We note that HDR efficiencies have been reported to be much higher than observed here [22, 25, 26]. Although we do not understand the basis for these differences, it might be attributable to the editing tools used, differences in ratios of donor versus nuclease-expressing vector used, and/or sensitivity of methodology used to score the genome editing events.

These results encouraged us to assess if chemical inhibition of DNA-PKcs would similarly affect HDR and/or NHEJ efficiency. We chose two well-characterized pharmacological inhibitors of DNA-PKcs, NU7441 [27] and KU-0060648 [28]. We first verified that both inhibitors were able to block DNA-PKcs kinase activity at non-toxic concentrations (Additional file 1: Figure S1A) following γ-irradiation of 293/TLR cells by monitoring phospho-H2AX level (Fig. 1e, compare lanes 3 and 4 to 1 and 2). To verify if these inhibitors could affect Cas9-mediated genome editing outcome, we transfected 293/TLR cells with Cas9 vectors expressing sgRNAs targeting either the neutral *Rosa26* locus or the TLR eGFP cistron and exposed cells to NU7441 or KU-0060648 16 h post transfection (Fig. 1b). As expected, no significant editing at the TLR locus was apparent in the presence of Cas9 and the *Rosa26* sgRNA (Fig. 1f). In contrast, the presence of Cas9 and the *sgGFP* yielded a significant proportion of mCherry^+^ cells due to NHEJ repair. Supplying a GFP repair template *in trans* resulted in approximately 2 % of the cells expressing GFP as a result of HDR-driven events (Fig. 1f – top right panel). The addition of NU7441 or KU-0060648 to cells following introduction of the Cas9/*sgGFP* editing system and the ΔGFP repair template caused a decrease of approximately 40 % in NHEJ events which was accompanied by an approximately two-fold stimulation in HDR (Fig. 1f, g). This effect was dose-dependent and reached a maximum at approximately 2.0 μM for NU7441 and 250 nM for KU-0060648 (Fig. 1g). Higher concentrations of either compounds were not well tolerated (Additional file 1: Figure S1). NU7441 or KU-0060648 are not selective for only DNA-PKcs, but also target PI3K, since both enzymes share highly homologous catalytic domains [29]. We therefore used siRNAs to suppress the PI3K-p110α catalytic subunit and found no evidence on genome editing outcome indicating that PI3K-p110α suppression is not mediating the effects of NU7441 or KU-0060648 (Fig. 1c, d).

**Fig. 2:**
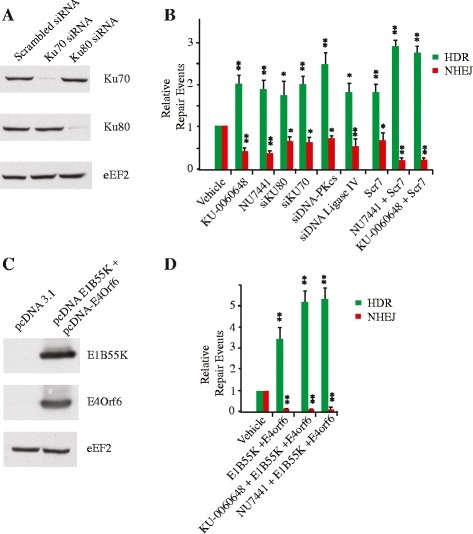
HDR stimulation by NU7441 and KU-0060648 is comparable to other HDR enhancing tools. **a** Western blot showing knock down efficiency obtained with the siRNAs targeting either Ku70 or Ku80. **b** Quantitation of genome editing events from cells transfected with pQCiG-TLR and ΔeGFP donor in the presence of 2 μM NU7441, 250 nM KU-0060648, siRNAs targeting Ku70, Ku80, DNA-PKcs or DNA ligase IV, 1 μM Scr7, or a combination of Scr7 and 2 μM NU7441 or 250 nM KU-0060648. The HDR and NHEJ values are relative to those obtained with Cas9, *sgRNA*, and ΔeGFP donor in the presence of vehicle (DMSO). Results are from biological replicates performed in technical duplicates. Significance (relative to vehicle) was calculated using the Student’s *t*-test: *p ≤0.05; **p ≤0.01; ns, not significant. **c** Western blot showing expression of Adenovirus 5 proteins E1B55K and E4orf6 following transfection into the 293/TLR line. **d** Quantitation of genome editing efficiencies was as in B except that adenovirus 5 proteins E1B55K and E4orf6 expression vectors were co-transfected with pQCiG-TLR and ΔeGFP donor plasmids and cultured in presence or absence of 2 μM NU7441 or 250 nM KU-0060648. *N* = 4; error bars represent S.D. Results are from biological replicates performed in technical duplicates. Significance (relative to vehicle) was calculated using the Student’s *t*-test: *p ≤0.05; **p ≤0.01; ns, not significant

We next wished to directly compare the efficiency of HDR stimulation by NU7441 and KU-0060648 to that of other tools recently reported to stimulate HDR. These include the DNA ligase IV inhibitor - Scr7, siRNAs to Ku70 and Ku80, and ectopic expression of the adenovirus 5 proteins E1B55K and E4orf6 [14, 15, 30]. Suppression of Ku70 and Ku80 by siRNAs in the 293/TLR line followed by introduction of Cas9/sgRNAs decreased NHEJ approximately 1.6-fold while stimulating HDR nearly two-fold (Fig. 2a and b) – a level similar to what we observed upon DNA-PK and DNA Ligase IV knockdown (Fig. 1c, d, and Fig. 2b). Treatment of 293/TLR cells with Scr7 showed a comparable effect on editing, namely a slight reduction in NHEJ and an approximately two-fold stimulation of HDR (Fig. 2b and Additional file 1: Figure S1A). Co-transfection of vectors expressing the adenovirus 5 proteins E1B55K and E4orf6 was the most efficient approach to inhibiting NHEJ (eight-fold reduction) while stimulating HDR 3.5-fold (Fig 2c, d) as previously reported [14]. Combining the DNA-PKcs inhibitors with either Scr7 or E1B55K/E4orf6 showed an additive effect on HDR (Fig. 2b and d).

**Fig. 3:**
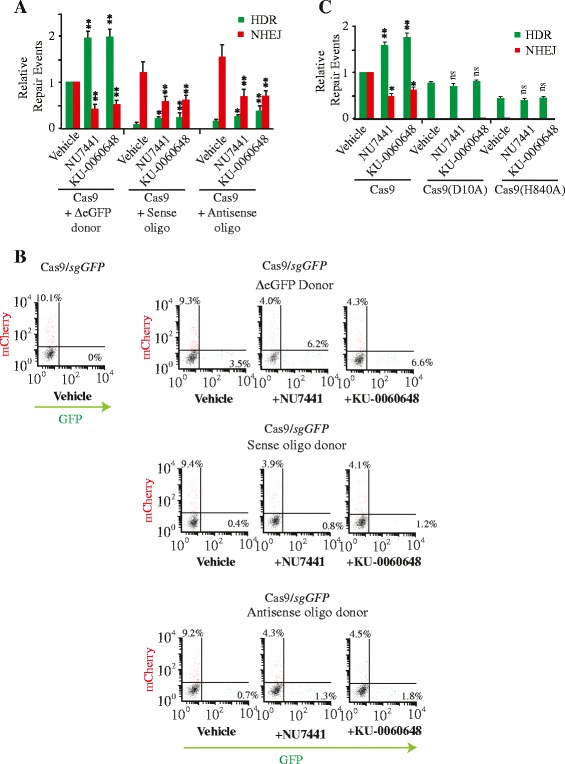
HDR directed by oligonucleotide donors are stimulated by DNA-PKcs inhibitors. **a** Quantitation of genome editing events from cells transfected with pQCiG-TLR and either ΔeGFP donor (2.5 kbp homology upstream and 1.5 kbp homology downstream of target site) or oligonucleotides (sense or antisense) (110 nucleotides) spanning the *sgGFP* target site and exposed to vehicle, 2 μM NU7441, or 250 nM KU-0060648. The HDR and NHEJ values are relative to those obtained with Cas9, *sgRNA*, and ΔeGFP donor in the presence of vehicle (DMSO). *N* = 4; error bars represent S.D. Results are from biological replicates performed in technical duplicates. Significance (relative to vehicle) was calculated using the Student’s *t*-test: *p ≤0.05; **p ≤0.01; ns, not significant. **b** Representative examples of FACS plot obtained in (**a**). Note that mCherry^+^ cells are reporting on only one-third of all NHEJ events. **c** Quantitation of genome editing events from experiments performed as described in (a) except using WT Cas9 or the D10A and H840A nickase variants and the ΔeGFP donor. Values are set relative to editing frequencies observed in 293/TLR cells transfected with WT Cas9 expression vector and propagated in the presence of vehicle. *N* = 4; error bars represent S.D. Results are from biological replicates performed in technical duplicates. Significance (relative to vehicle) was calculated using the Student’s *t*-test: *p ≤0.05; **p ≤0.01; ns, not significant

Since oligonucleotides have become popular as templates for directed changes following induction of a DSB by Cas9 and other gene editing tools [2, 31] we sought to determine if NU7441 or KU-0060648 could also stimulate HDR mediated by these much shorter, single-stranded DNA templates. Both sense and antisense oligonucleotide (relative to the eGFP ORF) functioned as repair templates following DSB induction. We noted that the antisense oligonucleotide led to slightly better repair efficiencies. This strand bias for the antisense oligonucleotide is not well understood but has been previously discussed [32]. It may involve steric interference on the transcribed strand blocking oligonucleotide binding. We also noted that repair efficiencies with oligonucleotides were approximately 10-fold lower than what was obtained using a longer double-stranded DNA template (Fig. 3a). This difference could be due to variations in transfection efficiencies compared to the DNA template donor, preferences for longer donor templates for HDR, and/or stability issues. Nonetheless, treatment of 293/TLR cells with NU7441 or KU-0060648 lead to a three- to four-fold increase in HDR and an approximately two-fold decrease in NHEJ-mediated repair events (Fig. 3a, b). These results indicate that DNA-PKcs inhibitors can also be used to stimulate HDR by oligonucleotide donor templates while suppressing NHEJ events.

One strategy that avoids NHEJ mediated events altogether is the use Cas9 nickase mutants (for example, Cas9(D10A) and Cas9(H840A)) since DNA nicks are repaired by the non-mutagenic base excision repair (single-strand break repair pathway) process while supporting HDR [33–35]. We therefore asked whether DNA-PKcs inhibitors would also stimulate genomic repair induced by a DNA nick. As expected, Cas9(D10A) or Cas9(H840A) targeting the TLR reporter did not lead to mutagenic repair (Fig. 3c). The levels of HDR repair obtained with the nickases were lower (approximately two-fold) than obtained with Cas9 (Fig. 3c) and this process was not stimulated by either NU7441 or KU-0060648 (Fig. 3c).

**Fig. 4:**
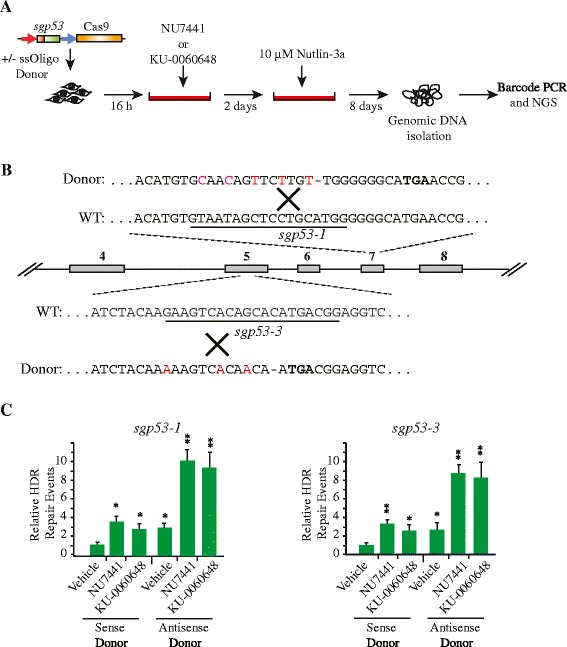
DNA-PK inhibitors stimulate homology-directed repair at the p53 locus. **a** Outline of experimental approach. **b** Position of target sites and partial sequence of oligonucleotides used as repair templates for p53 exons 5 and 7. A schematic representation of the murine p53 locus is shown with the locations of *sgp53*-1 and *sgp53*-3. The sequence of the donor templates for HDR (*sgp53*-1; 105 nucleotides, *sgp53*-3; 106 nucleotides) are indicated with red nucleotides denoting missense mutations and a dash indicating a frameshift mutation leading to a premature stop codon (in bold). **c** Fold stimulation of HDR at the *sgp53*-1 and *sgp53*-3 sites by NU7441 (2 μM) or KU-0060648 (250 nM). Results are shown as the fraction of all retrieved mutated sequences that correspond to HDR events in the presence of the DNA-PK inhibitors relative to vehicle. *N* = 4; error bars represent S.D. Results are from biological replicates performed in technical duplicates. Significance (relative to vehicle) was calculated using the Student’s *t*-test: *p ≤0.05; **p ≤0.01; ns, not significant

To determine if our results could be extended to an endogenous locus, we targeted p53 exons 5 and 7 using two previously characterized sgRNAs (*sgp53-3* and *sgp53-1*, respectively) [21]. Here, Cas9 and *sgp53-1* or *sgp53-3* were introduced into *Arf*^*−/−*^ mouse embryonic fibroblasts (MEFs) in the presence or absence of a corresponding oligonucleotide template designed to introduce a premature nonsense mutation (Fig. 4a). The oligonucleotides also harbored secondary silent mutations that enabled us to distinguish between nonsense mutations arising from HDR rather than fortuitously due to NHEJ (Fig. 4b). Following p53 targeting and exposure to NU7441 or KU-0060648, cells harboring p53 mutations were enriched in the presence of Nutlin-3a, an MDM2 antagonist and activator of p53 [21, 36] (Fig. 4a). Amplification, followed by sequencing of p53 exons 5 and 7, was used to determine the levels of donor-derived sequences in each sample (Fig. 4a). Approximately 800,000 reads were recovered from each target site, with approximately 78 % percent mapping back to the reference sequence. No mutations above background at either p53 exon were recovered from Cas9/*sgRosa26* transfected cells (data not shown). We observed approximately 1 % oligo-derived sequences from exons 5 and 7 in the absence of DNA-PKcs inhibitors and that, as in Fig. 3, the antisense oligonucleotide donor induced higher HDR correction efficiency compared to the sense donor oligonucleotide donor. As observed in the 293/TLR line, both NU7441 or KU-0060648 increased HDR approximately three-fold in the cell population (Fig. 4c). These results demonstrate that inhibition of DNA-PKcs can be used to stimulate HDR events at endogenous loci.

Stimulation of HDR following inhibition of DNA-PK with the small molecule IC86621 has been previously reported [20]. Our experiments extend these results using additional DNA-PKcs inhibitors by demonstrating their compatibility for use in conjunction with the Cas9 editing system (Fig. 1). In addition, we demonstrate that oligonucleotide-mediated HDR at endogenous loci is also stimulated by both NU7441 and KU-0060648 (Fig. 4). Although the increase in HDR obtained by treating cells with NU7441 or KU-0060648 was accompanied by a decrease in NHEJ, our experiments do not allow us to ascertain whether the stimulation of HDR is dependent on the concomitant decrease of NHEJ or reflects a direct inhibitory role of DNA-PKcs on HDR [20]. The fact that we did not observe a stimulation of HDR when nicks were introduced at the target loci (Fig. 3c) suggests an indirect effect. However, it has recently been reported that HDR mediated by nicks occurs via a pathway that is distinct from that induced by DSBs and is independent of RAD51 and BRCA2 [35]. Hence, one would expect a lack of response if the reported cross-talk between DNA-PKcs and HDR does not extend to the nickase-induced HDR pathway - an observation that will require further in-depth characterization. Although our work highlights the possibility to use DNA-PKcs inhibitors in order to stimulate HDR following Cas9-mediated cleavage, it should be kept in mind that inhibition of DNA-PKcs activity shunts some of the repair into alternate pathways [37], an event that could affect repair fidelity at double-stranded breaks that arise spuriously throughout the cell cycle.

It has been a significant challenge to obtain small molecule inhibitors that are selective to members of the PI3K family (PI3K, ATM, ATR, DNA-PK, and mTOR). KU-0060648 targets both DNA-PKcs and PI3K [28], whereas NU7441 shows 20-fold higher selective inhibition towards DNA-PK than PI3K in cells [38]. NU7441 appears to have little activity towards ATM and ATR [38]. Although we cannot formally ruled out that the results obtained herein are due to off-target effects of KU-0060648 and NU7441, our results are consistent with the observation that siRNA-mediated suppression of DNA-PK also stimulates HDR (Fig. 1d) [22]. Treatment of the 293/TLR reporter line with either rapamycin or PP242, two mTOR inhibitors, had no effect on NHEJ or HDR repair efficiencies, making it unlikely that KU0060648 and NU7441 are acting through inhibition of mTOR (data not shown). We also do not think that KU-0060648 and NU7441 are acting through inhibition of PI3K since siRNA-mediated suppression of PI3K did not phenocopy the effects of either compound (Fig. 1f).

## Conclusions

Our results indicate that transient pharmacological inhibition of DNA-PKcs can be used to stimulated HDR following Cas9-mediated induction of a DSB, thus enriching for HDR-mediated repair events. This is expected to reduce the downstream workload required to identify cells of interest having incorporated a desired, directed genomic modification.

## Additional file

Additional file 1: Figure S1. Toxicity of NU7441, KU-0060648 and Scr7 on the cell lines used in this study. **A** The 293/TLR line was exposed to increasing concentrations of NU7441, KU-0060648, and Scr7 for 5 days at which point the viability of the cells was determined. Viability is plotted relative to vehicle controls. *N* = 4; error bars represent S.D. Results are from biological replicates performed in technical duplicates. Significance (relative to vehicle) was calculated using the Student’s *t*-test: **P* ≤0.05; ***P* ≤0.01; ns, not significant. **B** Viability of *Arf* ^*−/−*^ MEFs exposed to NU7441 or KU-0060648. Experiments were performed as for 293/TLR cells in (A). (PDF 371 kb)

## Abbreviations

Cas: CRISPR associated protein 9
CRISPR: Clustered regularly interspaced short palindromic repeats
DNA-PKcs: DNA-dependent protein kinase catalytic subunit
DSB: Double-stranded break
eGFP: enhanced green fluorescent protein
GY: A Gray unit of ionization
HDR: Homology-directed recombination
MEFs: Murine embryonic fibroblasts
NHEJ: Non-homologous end joining
PAGE: Polyacrylamide gel electrophoresis
PAM: Protospacer adjacent motif
PI3K: Phosphoinositide 3-kinase
sgRNA: single-guide RNA
siRNA: Small interfering RNA
TLR: Traffic Light Reporter

## References

[1] Jinek M, Chylinski K, Fonfara I, Hauer M, Doudna JA, Charpentier E (2012). A programmable Dual-RNA-Guided DNA endonuclease in adaptive bacterial immunity. Science..

[2] Mali P, Yang L, Esvelt KM, Aach J, Guell M, DiCarlo JE (2013). RNA-guided human genome engineering via Cas9. Science..

[3] Cong L, Ran FA, Cox D, Lin S, Barretto R, Habib N (2013). Multiplex genome engineering using CRISPR/Cas systems. Science..

[4] Jinek M, East A, Cheng A, Lin S, Ma E, Doudna J (2013). RNA-programmed genome editing in human cells. eLife..

[5] Anders C, Niewoehner O, Duerst A, Jinek M (2014). Structural basis of PAM-dependent target DNA recognition by the Cas9 endonuclease. Nature..

[6] Lieber MR (2010). The mechanism of double-strand DNA break repair by the nonhomologous DNA end-joining pathway. Annu Rev Biochem..

[7] San Filippo J, Sung P, Klein H (2008). Mechanism of eukaryotic homologous recombination. Annu Rev Biochem..

[8] Dexheimer T, Mathews L, Cabarcas S, Hurt E (2013). DNA repair pathways and mechanisms. DNA repair of cancer stem cells.

[9] Mali P, Aach J, Stranges PB, Esvelt KM, Moosburner M, Kosuri S (2013). CAS9 transcriptional activators for target specificity screening and paired nickases for cooperative genome engineering. Nat Biotechnol..

[10] Wang H, Yang H, Shivalila CS, Dawlaty MM, Cheng AW, Zhang F (2013). One-step generation of mice carrying mutations in multiple genes by CRISPR/Cas-mediated genome engineering. Cell..

[11] Yang H, Wang H, Shivalila CS, Cheng AW, Shi L, Jaenisch R (2013). One-step generation of mice carrying reporter and conditional alleles by CRISPR/Cas-mediated genome engineering. Cell..

[12] Malina A, Mills JR, Cencic R, Yan Y, Fraser J, Schippers LM (2013). Repurposing CRISPR/Cas9 for in situ functional assays. Genes Dev..

[13] Pierce AJ, Hu P, Han M, Ellis N, Jasin M (2001). Ku DNA end-binding protein modulates homologous repair of double-strand breaks in mammalian cells. Genes Dev..

[14] Chu VT, Weber T, Wefers B, Wurst W, Sander S, Rajewsky K (2015). Increasing the efficiency of homology-directed repair for CRISPR-Cas9-induced precise gene editing in mammalian cells. Nat Biotechnol..

[15] Maruyama T, Dougan SK, Truttmann MC, Bilate AM, Ingram JR, Ploegh HL (2015). Increasing the efficiency of precise genome editing with CRISPR-Cas9 by inhibition of nonhomologous end joining. Nat Biotechnol..

[16] Rothkamm K, Kruger I, Thompson LH, Lobrich M (2003). Pathways of DNA double-strand break repair during the mammalian cell cycle. Mol Cell Biol..

[17] Lin S, Staahl BT, Alla RK, Doudna JA (2014). Enhanced homology-directed human genome engineering by controlled timing of CRISPR/Cas9 delivery. eLife..

[18] Yu C, Liu Y, Ma T, Liu K, Xu S, Zhang Y (2015). Small molecules enhance CRISPR genome editing in pluripotent stem cells. Cell Stem Cell..

[19] Allen C, Kurimasa A, Brenneman MA, Chen DJ, Nickoloff JA (2002). DNA-dependent protein kinase suppresses double-strand break-induced and spontaneous homologous recombination. Proc Natl Acad Sci U S A..

[20] Neal JA, Dang V, Douglas P, Wold MS, Lees-Miller SP, Meek K (2011). Inhibition of homologous recombination by DNA-dependent protein kinase requires kinase activity, is titratable, and is modulated by autophosphorylation. Mol Cell Biol..

[21] Cencic R, Miura H, Malina A, Robert F, Ethier S, Schmeing TM (2014). Protospacer adjacent motif (PAM)-distal sequences engage CRISPR Cas9 DNA target cleavage. PLoS One..

[22] Certo MT, Ryu BY, Annis JE, Garibov M, Jarjour J, Rawlings DJ (2011). Tracking genome engineering outcome at individual DNA breakpoints. Nat Methods..

[23] Gratz SJ, Ukken FP, Rubinstein CD, Thiede G, Donohue LK, Cummings AM (2014). Highly specific and efficient CRISPR/Cas9-catalyzed homology-directed repair in Drosophila. Genetics..

[24] Beumer KJ, Trautman JK, Bozas A, Liu JL, Rutter J, Gall JG (2008). Efficient gene targeting in Drosophila by direct embryo injection with zinc-finger nucleases. Proc Natl Acad Sci U S A..

[25] Hendel A, Kildebeck EJ, Fine EJ, Clark JT, Punjya N, Sebastiano V (2014). Quantifying genome-editing outcomes at endogenous loci with SMRT sequencing. Cell Rep..

[26] Voit RA, Hendel A, Pruett-Miller SM, Porteus MH (2014). Nuclease-mediated gene editing by homologous recombination of the human globin locus. Nucleic Acids Res..

[27] Leahy JJ, Golding BT, Griffin RJ, Hardcastle IR, Richardson C, Rigoreau L (2004). Identification of a highly potent and selective DNA-dependent protein kinase (DNA-PK) inhibitor (NU7441) by screening of chromenone libraries. Bioorg Med Chem Lett..

[28] Munck JM, Batey MA, Zhao Y, Jenkins H, Richardson CJ, Cano C (2012). Chemosensitization of cancer cells by KU-0060648, a dual inhibitor of DNA-PK and PI-3K. Mol Cancer Ther..

[29] Liu P, Cheng H, Roberts TM, Zhao JJ (2009). Targeting the phosphoinositide 3-kinase pathway in cancer. Nat Rev Drug Discov..

[30] Cheng CY, Gilson T, Dallaire F, Ketner G, Branton PE, Blanchette P (2011). The E4orf6/E1B55K E3 ubiquitin ligase complexes of human adenoviruses exhibit heterogeneity in composition and substrate specificity. J Virol..

[31] Chen F, Pruett-Miller SM, Huang Y, Gjoka M, Duda K, Taunton J (2011). High-frequency genome editing using ssDNA oligonucleotides with zinc-finger nucleases. Nat Methods..

[32] Jensen NM, Dalsgaard T, Jakobsen M, Nielsen RR, Sorensen CB, Bolund L (2011). An update on targeted gene repair in mammalian cells: methods and mechanisms. J Biomed Sci..

[33] Katyal S, McKinnon PJ (2011). Disconnecting XRCC1 and DNA ligase III. Cell Cycle..

[34] Lee GS, Neiditch MB, Salus SS, Roth DB (2004). RAG proteins shepherd double-strand breaks to a specific pathway, suppressing error-prone repair, but RAG nicking initiates homologous recombination. Cell..

[35] Davis L, Maizels N (2014). Homology-directed repair of DNA nicks via pathways distinct from canonical double-strand break repair. Proc Natl Acad Sci U S A..

[36] Tovar C, Rosinski J, Filipovic Z, Higgins B, Kolinsky K, Hilton H (2006). Small-molecule MDM2 antagonists reveal aberrant p53 signaling in cancer: implications for therapy. Proc Natl Acad Sci U S A..

[37] Potts PR, Porteus MH, Yu H (2006). Human SMC5/6 complex promotes sister chromatid homologous recombination by recruiting the SMC1/3 cohesin complex to double-strand breaks. EMBO J..

[38] Tavecchio M, Munck JM, Cano C, Newell DR, Curtin NJ (2012). Further characterisation of the cellular activity of the DNA-PK inhibitor, NU7441, reveals potential cross-talk with homologous recombination. Cancer Chemother Pharmacol..

[39] Donnelly ML, Hughes LE, Luke G, Mendoza H, ten Dam E, Gani D (2001). The ‘cleavage’ activities of foot-and-mouth disease virus 2A site-directed mutants and naturally occurring ‘2A-like’ sequences. J Gen Virol..

[40] Donnelly ML, Luke G, Mehrotra A, Li X, Hughes LE, Gani D (2001). Analysis of the aphthovirus 2A/2B polyprotein ‘cleavage’ mechanism indicates not a proteolytic reaction, but a novel translational effect: a putative ribosomal ‘skip’. J Gen Virol..

