# Supplementary figures and images for: Pharmacological inhibition of DNA-PK stimulates Cas9-mediated genome editing
Source: Genome Med. 2015 Aug 27;7(1):93. doi: 10.1186/s13073-015-0215-6 (PMC4550049; doi:10.1186/s13073-015-0215-6)

**A** 293/TLR

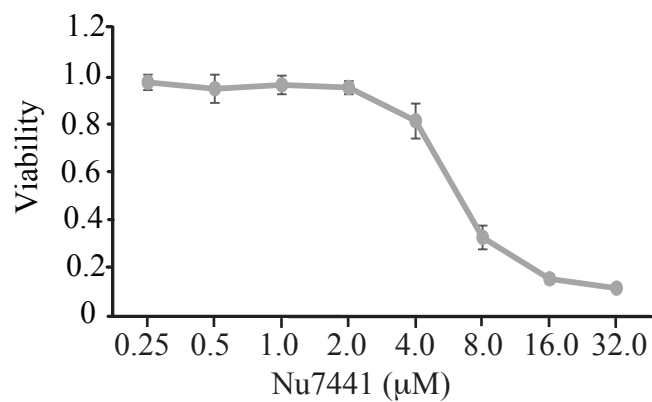

**B** *Arf*<sup>-/-</sup> MEFs

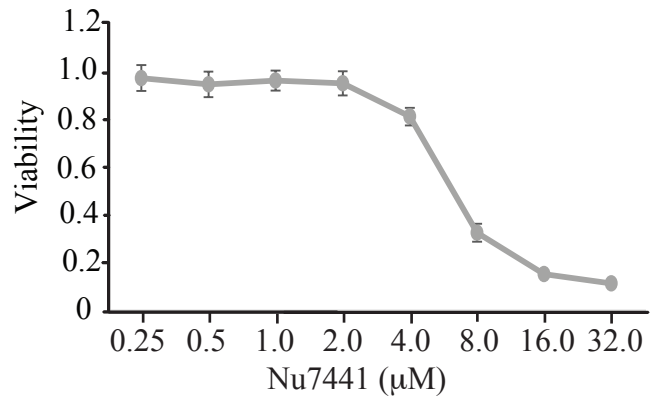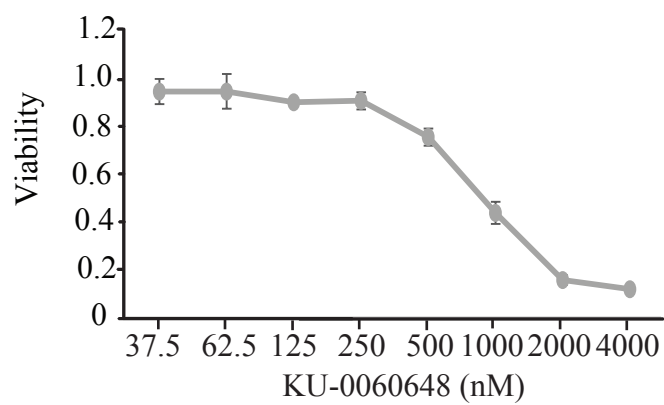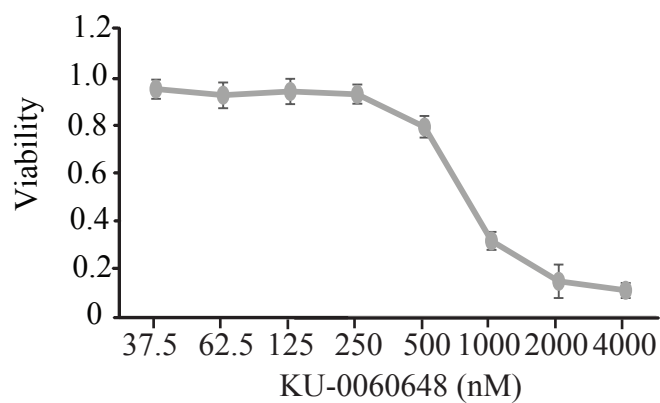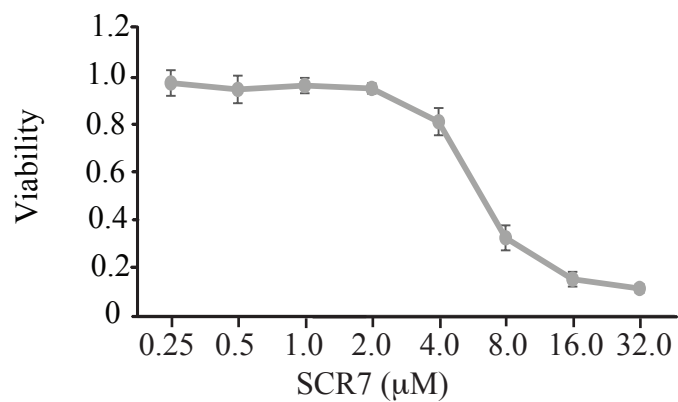

Supplement: Additional file 1: Figure S1. — Toxicity of NU7441, KU-0060648 and Scr7 on the cell lines used in this study. A The 293/TLR line was exposed to increasing concentrations of NU7441, KU-0060648, and Scr7 for 5 days at which point the viability of the cells was determined. Viability is plotted relative to vehicle controls. N = 4; error bars represent S.D. Results are from biological replicates performed in technical duplicates. Significance (relative to vehicle) was calculated using the Student’s t-test: *P ≤0.05; **P ≤0.01; ns, not significant. B Viability of Arf −/− MEFs exposed to NU7441 or KU-0060648. Experiments were performed as for 293/TLR cells in (A). (PDF 371 kb) [file 13073_2015_215_MOESM1_ESM.pdf]
